# Selected Research Articles from the 2019 International Workshop on Computational Network Biology: Modeling, Analysis, and Control (CNB-MAC)

**DOI:** 10.1186/s12864-020-06934-y

**Published:** 2020-09-07

**Authors:** Byung-Jun Yoon, Xiaoning Qian, Tamer Kahveci, Ranadip Pal

**Affiliations:** 1 Department of Electrical and Computer Engineering, Texas A&M University, College Station, TX 77843 USA; 2 TEES-AgriLife Center for Bioinformatics and Genomic Systems Engineering (CBGSE), College Station, TX 77845 USA; 3 Computational Science Initiative, Brookhaven National Laboratory, Upton, NY 11973 USA; 4 Department of Computer and Information Science and Engineering, University of Florida, Gainesville, FL 32611-6125 USA; 5 Department of Electrical and Computer Engineering, Texas Tech University, Lubbock, TX 79409−3102 USA

## Introduction

The 6th International Workshop on Computational Network Biology: Modeling, Analysis, and Control (CNB-MAC 2019) was held in Niagara Falls, New York, on September 7, 2019. The workshop was organized in conjunction with the 10th ACM Conference on Bioinformatics, Computational Biology, and Health Informatics (ACM-BCB), the flagship conference of the ACM SIGBio. The CNB-MAC workshop aims to provide an international scientific forum for presenting recent advances in computational network biology that involve modeling, analysis, and control of biological systems and system-oriented analysis of large-scale OMICS data.

CNB-MAC 2019 was co-chaired by Drs. Byung-Jun Yoon, Xiaoning Qian, Tamer Kahveci, and Ranadip Pal. The workshop featured original research papers [1–10], a highlight presentation of a recently published journal paper [11], and poster presentations [12, 13], which were selected by the workshop chairs based on the reviews performed by the technical committee members. Reports from previous CNB-MAC workshops are available at [14–16].

Thanks to the generous support provided by the National Science Foundation (NSF), Student Travel Grants have been awarded to student authors of outstanding research papers and posters that have been invited for presentation at CNB-MAC 2019. The Travel Grants have also supported several minority students who do not have a presentation at the workshop, in order to promote diversity. Dr. Ranadip Pal served as the award chair for CNB-MAC 2019. Seventeen awardees were selected by the award committee after a careful review of the applications and the submitted work.

## Research papers presented at CNB-MAC 2019

After the workshop, ten original research papers [1–10] were accepted for publication in the CNB-MAC 2019 partner journals: *BMC Bioinformatics* and *BMC Genomics*. In the following we provide a brief summary of these selected papers.

Haplotypes, the ordered lists of single nucleotide variations that distinguish chromosomal sequences from their homologous pairs, may reveal an individual’s susceptibility to hereditary and complex diseases and affect how our bodies respond to therapeutic drugs. Reconstructing haplotypes of an individual from short sequencing reads is an NP-hard problem that becomes even more challenging in the case of polyploids. While increasing lengths of sequencing reads and insert sizes helps improve accuracy of reconstruction, it also exacerbates computational complexity of the haplotype assembly task. This has motivated the pursuit of algorithmic frameworks capable of accurate yet efficient assembly of haplotypes from high-throughput sequencing data. Sankararaman, Vikalo, and Baccelli [1] propose a novel graphical representation of sequencing reads and pose the haplotype assembly problem as an instance of community detection on a spatial random graph. To this end, a spatial graph where each read is a node with an unknown community label associating the read with the haplotype it samples from is constructed. Haplotype reconstruction is then achieved through a two-step procedure: first, the community labels on the nodes (i.e., the reads) are recovered, and then these estimated labels are used to assemble the haplotypes. Based on this observation, ComHapDet a novel assembly algorithm for diploid and ployploid haplotypes is developed which allows both bialleleic and multi-allelic variants.

B cell affinity maturation is a microevolution process that enables the immune system to generate high-affinity antibodies and develop high diversity of the immunoglobulin repertoires. This microevolution process can be described by lineage trees constructed from BCR (B cell immunoglobulin receptor) sequencing data. Yang et al. [2] present a novel algorithm named GLaMST (Grow Lineages along Minimum Spanning Tree) for constructing such lineage trees. Through simulated and real data, GLaMST is shown to outperform existing algorithms in both efficiency and accuracy. Integrating GLaMST into existing BCR sequencing analysis frameworks can significantly improve the lineage tree reconstruction aspect BCR sequencing analysis.

Lee and Kimmel [3] propose that G-Networks and Stochastic Automata Networks (SANs), are useful to identify a set of genes that play an important role in a system of interest and to estimate their correlation. Their study uses G-Networks stationary and transient distributions to detect statistically significant genes associated with telomere maintenance mechanisms (TMMs), essential for immortalization of cell populations. A new algorithm based on SANs is introduced to show how the correlation between two genes of interest varies in the transient state with different TMM and different cell condition. This analysis expands knowledge of details of genetic control of the TMMs.

In [4], Dadaneh et al. propose a fully generative hierarchical gamma-negative binomial (hGNB) model for extracting low-dimensional representations of single-cell RNA sequencing (scRNA-seq) data. The proposed hGNB model can naturally account for covariate effects at both gene and cell levels to identify complex latent representations of scRNA-seq data, without the need for explicitly modeling zero inflation in scRNA-seq data or commonly adopted pre-processing steps including normalization in many existing methods. By exploiting conditional conjugacy via novel data augmentation techniques, hGNB possesses efficient Bayesian model inference with closed-form Gibbs sampling update equations. Experimental results on both simulated data and several real-world scRNA-seq datasets show that hGNB is a powerful tool for cell cluster discovery as well as cell lineage inference.

Progression of the cell cycle in *C. crescentus* requires precise coordination of metabolic and morphological cell activities. The guanine nucleotide-based messenger network, including c-di-GMP and (p) ppGpp, plays significant roles in controlling metabolisms and morphology, such as regulating the activity of CtrA, deciding transition between motile and non-motile cells, and adapting cells to environmental changes. Xu et al. [5] propose a mathematical model for *C. crescentus* to capture the dynamics of c-di-GMP and (p) ppGpp and relate the second messenger network with environmental response through a nitrogen PTS system. Their simulations are consistent with experimental observations and suggest potential pathways about nutrient availability influencing cell cycle of *C. crescentus*.

The identification of essential genes in bacteria not only allows life scientists to determine the set of genes that are critical for the survival of an organism, it can also provide targets for antimicrobial/antibiotic drugs and the creation of self-sustaining artificial genomes. DeeplyEssential [6] leverages a deep neural network architecture for the identification of bacterial essential genes exclusively from the primary DNA sequence, thus maximizing the practicality of the tool.

The advent of single-cell Hi-C brings a new type of frequency information, the number of single cells with chromatin interactions between two disjoint chromosome regions, which is ignored in research on interchromosomal interactions. Bulathsinghalage and Liu [7] propose a computational tool to identify regions with statistically frequent interchromosomal interactions at single-cell resolution. They demonstrate that the tool utilizing networks and binomial statistical tests can identify interesting structural regions through visualization, comparison and enrichment analysis and it also supports different configurations to provide users with flexibility.

TCGA (The Cancer Genome Atlas) is a wonderful data resource for developing algorithms and models toward better understanding of cancers. Clayton et al. [8] integrate gene expression data, drug treatment data, and patient survival data in TCGA, and develop machine learning models to predict whether a patient will respond positively or negatively to two chemotherapeutics: 5-Fluorouracil and Gemcitabine. Results show prediction accuracies of up to 86%, and the most informative genes for the models are enriched in well-known cancer signaling pathways. Overall, this integrative analysis demonstrates the utility of drug treatment data, which is an under-explored aspect compared to other genomic aspects available through TCGA.

Zengin and Önal-Süzek [9] propose a reusable and open-source R pipeline for the discovery of prognostic signatures by the integration of multiple dimensions of TCGA Lung cancer (LUAD) dataset. The authors generate 4 different gene categories using the significant SNVs, CNVs, DEGs and active subnetwork DEGs. Multivariate Cox proportional hazards model with the Lasso penalty and LOOCV is used to identify the best gene signature among the gene categories. The authors elucidate a 12-gene signature (BCHE, CCNA1, CYP24A1, DEPTOR, MASP2, MGLL, MYO1A, PODXL2, RAPGEF3, SGK2, TNNI2, ZBTB16) for prognostic risk prediction based on overall survival time of the patients with lung adenocarcinoma. When the patients are clustered into high-risk and low-risk groups with the proposed framework, the survival analysis show highly significant results for both training (55 TCGA LUAD patients) and test (442 TCGA LUAD patients) datasets.

While many studies have attempted to combine gene network information with gene expression for predicting cancer outcomes, the issue of whether such combination actually provides more accurate prediction and identifies more robust biomarkers is complex due to the sophisticated experimental setup of different studies. Adnan et al. [10] propose a simple edge-based model to predict breast cancer metastasis using protein-protein interaction and gene co-expression networks. Using multiple evaluation metrics on 12 breast cancer patient cohorts, their rigorous evaluation shows that edge-based prediction performs consistently better than gene expression alone in random forest and logistic regression classifiers, and that the simple method outperforms several existing network-based methods with statistical significance. In addition, with a novel procedure to obtain important features from random forest models, they show that edge features are much more robust than gene features and the top biomarkers from edge features are statistically more significantly enriched in biological processes that are well known to be related to breast cancer metastasis.

## Data Availability

Not applicable.
